# Development and initial validation of the perceived career barrier scale for midwifery undergraduates in China (PCBS-MU): a cross-sectional instrument development study

**DOI:** 10.1186/s12912-026-04997-y

**Published:** 2026-07-07

**Authors:** Yue Li, Rui Yang, Na Wang, Xiaoxiao Ding, Nanxi Kang, Xinfen Xu

**Affiliations:** 1 Beijing Obstetrics and Gynecology Hospital, Capital Medical University, Beijing Maternal and Child Health Care Hospital, Beijing, China; 2 School of Nursing, Capital Medical University, Beijing, China; 3 Women’s Hospital, School of Medicine, Zhejiang University, Hangzhou, Zhejiang China

**Keywords:** Career barriers, Midwifery education, Scale development, Parenting style, China, Nursing students

## Abstract

**Background:**

Midwifery education in China faces growing retention challenges, yet no validated instrument for assessing perceived career barriers exists for this population. Existing measures, such as the Career Barriers Inventory, were developed for Western adult working populations. They do not capture profession-specific obstacles relevant to Chinese midwifery undergraduates, including concerns about labour market conditions, clinical competence demands, and the influence of family socialisation norms on career decisions. This gap limits the ability to design targeted educational interventions. This study therefore aimed to develop and test a self-report tool measuring perceived career barriers among Chinese midwifery undergraduates, and to examine the impact of family socialisation environment on their perceived career barriers.

**Methods:**

A total of 767 midwifery undergraduates from eight universities across five regions of China completed the Perceived Career Barrier Scale for Midwifery Undergraduates (PCBS-MU), a 33-item instrument developed through literature review, expert consultation (content validity index ≥ 0.78), and cognitive interviews. Exploratory factor analysis (EFA) with parallel analysis, internal consistency, and corrected item-total correlations were computed. Confirmatory factor analysis (CFA) with modification indices was conducted to evaluate structural validity. Kruskal-Wallis and Mann-Whitney U tests, hierarchical linear regression, and Spearman correlations examined demographic associations.

**Results:**

EFA supported a single underlying factor (KMO = 0.962; Bartlett’s χ²(528) = 23185.95, *p* < .001; eigenvalue = 16.59, explaining 50.3% of variance; factor loadings 0.50–0.87). Internal consistency was excellent (α = 0.968; corrected item-total correlations 0.50–0.78). CFA with six pairs of within-domain correlated residuals demonstrated good structural validity (χ²/df = 4.38, CFI = 0.930, TLI = 0.917, RMSEA = 0.074, SRMR = 0.068). Parenting style was the strongest predictor. Compared with authoritative parenting, authoritarian (B = 11.03), permissive (B = 6.24), and uninvolved (B = 12.63) styles were independently associated with higher barrier scores (all *p* < .001).

**Conclusions:**

The PCBS-MU is a unidimensional measure of perceived career barriers for Chinese midwifery undergraduates with satisfactory initial psychometric properties. In an exploratory secondary analysis, family socialisation environment, particularly parenting style, emerged as the dominant correlate of career barrier perceptions, suggesting that educational interventions targeting psychological resources may help reduce attrition in this workforce-critical group.

## Introduction

Midwifery is recognized internationally as a cornerstone of safe childbirth and maternal health. The World Health Organization (WHO) estimated a global deficit of approximately 900,000 midwives, a gap projected to close only partially by 2030 [1]. In China, the workforce pressure has intensified since the relaxation of the one-child policy, which led to a marked increase in births among older and higher-risk mothers, greater demand for caesarean deliveries, and a broader need for skilled midwifery care [2]. Consequently, retaining students through undergraduate midwifery training has become a critical workforce priority.

The measurement of perceived career barriers among Chinese midwifery undergraduates is further complicated by a structural feature unique to this population: a disconnected professional licensing pathway. Although midwifery in China is offered as a four-year undergraduate programme, graduates are not licensed directly as midwives upon completion. Instead, they must first pass the national registered nurse examination [3, 4], secure at least one year of employment in an obstetric setting, and only then become eligible to sit the maternal and infant health care licensing examination [5, 6]. This sequential, cross-qualification requirement means that midwifery graduates enter a prolonged period of professional ambiguity — holding nursing credentials while pursuing a midwifery career — which may heighten perceptions of career uncertainty and institutional barrier. Compounded by the profession’s high clinical demands, including high-stakes intrapartum decision-making, continuous emotional labour, and physically intensive shift work, this pathway creates a set of perceived career barriers qualitatively distinct from those faced by general nursing students, and not captured by instruments developed for other populations [7, 8].

Perceived career barriers are defined as an individual’s subjective appraisal that internal or external obstacles stand between themselves and desired career goals [9]. In nursing and midwifery contexts, elevated barrier perceptions have been associated with reduced professional commitment [10], weaker professional identity [11, 12], and higher rates of attrition [13].

Despite this evidence, research on career barriers in midwifery remains sparse, and existing instruments have significant limitations when applied to midwifery student populations. The Career Barriers Inventory (CBI) [9] and its adaptations [14] were developed primarily for Western adult working populations and therefore do not capture the profession-specific obstacles relevant to Chinese midwifery undergraduates, including concerns about medium- to long-term labour market conditions (e.g., declining birth rates and shrinking obstetric caseloads), high professional competence demands, and the influence of Chinese family socialisation norms that often favour more prestigious professions over midwifery [15]. To our knowledge, no validated scale exists specifically for Chinese midwifery undergraduates, and the few available studies in this population have relied on instruments adapted from general nursing contexts or on unvalidated measures, limiting cross-study comparisons and the ability to design targeted educational interventions [16, 17].

Drawing on Social Cognitive Career Theory (SCCT) [18], as an organising framework, the present study posits that contextual background factors, such as family socialisation environment, influence career-related cognitions through internal psychological mechanisms, including self-efficacy beliefs and coping strategies. From this perspective, parenting style, as a key dimension of family socialisation, should shape how students appraise professional obstacles [19]. Less supportive parenting styles, particularly authoritarian and uninvolved styles, which are characterised by low responsiveness and limited encouragement of autonomous decision-making, are therefore hypothesised to be associated with higher perceived career barriers, as they provide fewer psychological resources for navigating occupational uncertainty [15, 19].

The present study therefore had two aims: (1) to develop and validate a career barrier scale tailored to Chinese midwifery undergraduates, the Perceived Career Barrier Scale for Midwifery Undergraduates (PCBS-MU), following established best-practice guidelines for instrument development [20]; and (2) to conduct a prespecified exploratory analysis examining associations between demographic and family background variables and PCBS-MU scores, with particular attention to parenting style as a theoretically derived predictor grounded in SCCT. The findings are expected to provide educators and policymakers with a psychometrically grounded tool and evidence-based targets for reducing career barrier perceptions and supporting retention in this workforce-critical population.

## Methods

### Design and setting

This cross-sectional, multisite study was conducted between January and March 2025. Eight universities spanning five geographic regions of China (Northeast, East, South, Southwest, and North) were selected purposively to ensure regional coverage. All enrolled midwifery undergraduates at each institution were invited to participate.

### Participants

Students were eligible if they were enrolled in a four-year midwifery undergraduate programme and willing to participate. Based on a minimum of 20 observations per predictor variable for regression analyses [21], a minimum sample of 180 was targeted. The final sample comprised 767 participants (response rate 98.4% of 779 distributed). Twelve questionnaires were excluded prior to analysis: ten failed an embedded attention-check item and two were withdrawn by participants before submission. No missing data were present in the retained sample. This sample size also satisfies recommended thresholds for exploratory factor analysis: with 767 participants and 33 items, the participant-to-item ratio of 23:1 exceeds the minimum of 10:1 recommended for stable factor solutions [22], and falls within the “excellent” range (≥ 500) identified by Kaiser [23].

### Instrument development

Scale development followed established best-practice procedures for instrument construction [20]. Item generation drew on a systematic review of existing career barrier instruments, principally the Career Barriers Inventory [9] and several Chinese nursing career scales, with item wording modified to reflect the specific context of midwifery undergraduate training. An initial pool of 48 items was generated to represent seven theoretically derived content domains: institutional guidance deficit, career development constraints, career exploration uncertainty, practical competence concerns, psychological resilience barriers, family and peer obstacles, and personal attribute concerns. These seven domains were informed by SCCT’s tripartite framework: institutional guidance deficit, career development constraints, and career exploration uncertainty reflect contextual barriers to learning and performance; practical competence concerns and psychological resilience barriers correspond to self-efficacy determinants; and family and peer obstacles and personal attribute concerns represent proximal and distal background contextual factors as conceptualised within SCCT [18].

Content validity was assessed through expert review. Six senior midwifery educators independently rated each item for relevance on a four-point scale (1 = not relevant, 4 = highly relevant). Items with an item-level content validity index (I-CVI) below 0.78 were revised or removed [24], reducing the pool to 33 items. The expert review was conducted in a single round; items meeting the I-CVI threshold were retained without further revision. Cognitive interviews with 30 volunteer midwifery students confirmed that all retained items were understood as intended. The final PCBS-MU comprises 33 items rated on a five-point Likert scale (1 = strongly disagree to 5 = strongly agree), with higher scores indicating greater perceived barriers; the possible total score range is 33–165.

### Demographic questionnaire

A self-developed questionnaire collected ten variables: sex, academic grade (year 1–4), clinical placement experience (observation only, internship, or none), ethnicity, province of origin, urban or rural family background, only-child status, university attended, student leadership role, and parenting style. Parenting style was assessed using a single-item measure based on Baumrind’s four-category typology [25] (authoritative, authoritarian, permissive, or uninvolved), with brief descriptors for each style, consistent with established survey practice in Chinese nursing education research.

### Ethical considerations and data collection

Ethical approval was granted by the Ethics Committee of Capital Medical University (No. Z2024SY066). All participants provided electronic informed consent before accessing the questionnaire; no personally identifiable data were collected. Programme coordinators at each university distributed a survey link or QR code via WeChat. The platform restricted submissions to one per device and IP address to prevent duplicate entries. An embedded attention-check item was used to identify inattentive respondents. Completion of all items was required by the platform; partial submissions were therefore not possible. The survey was open for four weeks; a single reminder was sent at week two. DeepSeek was used for language editing of the manuscript.

### Data analysis

All analyses were conducted in IBM SPSS Statistics 26.0 (IBM Corp., 2019). Normality of total scores was assessed with the Shapiro-Wilk test; given significant departure from normality (W = 0.974, *p* < .001), non-parametric tests were used for group comparisons. Mann-Whitney U tests compared two-group variables (sex, residence, only-child status, student leadership role); Kruskal-Wallis H tests compared three or more groups (grade, clinical experience, parenting style). Effect sizes were calculated as rank-biserial r for Mann-Whitney and eta-squared (η²) for Kruskal-Wallis. Bonferroni-corrected pairwise comparisons were conducted where the Kruskal-Wallis test was significant.

Psychometric analyses included exploratory factor analysis (EFA) using maximum likelihood estimation with parallel analysis (200 random datasets; 95th percentile criterion) to determine the number of factors to retain. The Kaiser-Meyer-Olkin (KMO) measure and Bartlett’s test of sphericity assessed the suitability of the data for factor analysis. Internal consistency was evaluated with Cronbach’s α, and item quality was assessed using corrected item-total correlations (threshold ≥ 0.30). Confirmatory factor analysis (CFA) was conducted in AMOS 28.0. Model fit was evaluated using χ²/df, CFI, TLI, RMSEA, and SRMR. Where modification indices indicated large residual covariances between items within the same content domain, correlated residuals were added if a substantive theoretical rationale could be provided.

Hierarchical linear regression identified independent demographic predictors of PCBS-MU total scores, with parenting style entered as dummy-coded variables (authoritative as the reference category). Multicollinearity was evaluated using variance inflation factors (VIF; threshold < 10). Spearman’s rank correlations examined bivariate associations between significant predictors and total scores. Statistical significance was set at α = 0.05 throughout, with Bonferroni correction applied for pairwise post-hoc comparisons. Pearson correlations between content domain mean scores were additionally computed as supplementary evidence for the single-factor structure, to examine the degree of inter-domain overlap. Associations between demographic and family background variables and PCBS-MU scores were examined as a prespecified exploratory analysis to generate hypotheses for future validation studies.

## Results

### Sample characteristics

The sample of 767 participants was predominantly female (*n* = 735, 95.8%), consistent with the demographic profile of midwifery programmes in China. Academic year was distributed across year 1 (*n* = 182, 23.7%), year 2 (*n* = 156, 20.3%), year 3 (*n* = 238, 31.0%), and year 4 (*n* = 191, 24.9%). Just over half (*n* = 441, 57.5%) came from rural family backgrounds, and 490 participants (63.9%) had some form of clinical placement experience (observation or internship). Regarding parenting style, the majority reported authoritative parenting (*n* = 387, 50.5%), followed by permissive (*n* = 254, 33.1%), authoritarian (*n* = 73, 9.5%), and uninvolved (*n* = 53, 6.9%). Participant characteristics are summarised in Table 1.

**Table 1:** Participant characteristics and PCBS-MU score comparisons (*N* = 767)

Characteristic	*n* (%)	Mdn	IQR	Test statistic	*p*	ES
Sex				U = 10,867	0.467	*r* = .08
Female	735 (95.8)	102.0	[90, 116]			
Male	32 (4.2)	99.5	[83, 115]			
Grade				H(3) = 6.69	0.082	η² = 0.005
Year 1	182 (23.7)	99.0	[85, 114]			
Year 2	156 (20.3)	104.5	[96, 120]			
Year 3	238 (31.0)	100.0	[89, 114]			
Year 4	191 (24.9)	103.0	[87, 115]			
Clinical experience				H(2) = 2.04	0.360	η² = 0.000
Observation only	291 (37.9)	99.0	[88, 114]			
Internship	199 (26.0)	103.0	[87, 115]			
None	277 (36.1)	103.0	[91, 117]			
Residence				U = 62,580	0.002	*r* = .13
Urban	326 (42.5)	99.0	[87, 113]			
Rural	441 (57.5)	104.0	[91, 117]			
Only child				U = 45,136	0.103	*r* = .08
Yes	163 (21.2)	99.0	[86, 113]			
No	604 (78.8)	102.5	[90, 116]			
Student leadership role				U = 64,090	0.889	*r* = .01
Yes	249 (32.5)	103.0	[87, 116]			
No	518 (67.5)	101.0	[90, 116]			
Parenting style				H(3) = 29.87	< 0.001	η² = 0.035
Authoritative	387 (50.5)	99.0	[86, 112]			
Authoritarian	73 (9.5)	109.0	[96, 127]			
Permissive	254 (33.1)	104.0	[96, 118]			
Uninvolved	53 (6.9)	110.0	[97, 123]			
Total PCBS-MU		102.0	[90, 116]			
Mean (SD)	101.93 (24.55)					

Note. Mdn = median; IQR = interquartile range; ES = effect size (rank-biserial r for U tests; η² for H tests). Significance level α = 0.05. p values for parenting style and residence survive Bonferroni correction where applicable

### PCBS-MU total score distribution

Total PCBS-MU scores ranged from 33 to 165 (theoretical range 33–165), with 17 participants at the scale floor and 16 at the ceiling. The distribution was non-normal (W = 0.974, *p* < .001), with a median of 102.0 (IQR: 89.5–116.0), mean of 101.93 (SD = 24.55). The mean item score was 3.09 (SD = 1.10) on the five-point scale, indicating moderate levels of perceived career barriers overall. Descriptive statistics for the overall scale are summarised in Table 1.

### Exploratory factor analysis

EFA suitability was confirmed by KMO = 0.962 (excellent) and Bartlett’s test of sphericity (χ²(528) = 23185.95, *p* < .001). Parallel analysis indicated that only the first factor had an eigenvalue (16.59) substantially exceeding the 95th percentile of eigenvalues from random data (1.46), supporting a single-factor solution. The second (2.44) and subsequent factors fell below or near the parallel analysis threshold. The single factor explained 50.3% of the total variance (53.7% when estimated by maximum likelihood), with all 33 items loading substantially on this factor (loadings 0.50–0.87). Factor loadings are presented in Table 2.

The large first eigenvalue relative to subsequent eigenvalues (ratio 16.59:2.44 = 6.8:1) and the uniform pattern of item loadings are consistent with a single dominant latent construct: a global appraisal of perceived career barriers in midwifery. Although the 33 items were initially organised into seven content domains during item development, the EFA indicates that these domains reflect facets of a single overarching construct rather than empirically distinct dimensions. This is consistent with the high inter-domain correlations observed (*r* = .42–0.79) and the exceptionally high overall internal consistency (α = 0.968).

**Table 2:** PCBS-MU item content, factor loadings, and item-total correlations

No.	Item content (abbreviated)	Factor loading	ITC	Mean (SD)
	**Domain A: Institutional guidance deficit**			
A1	Career guidance and counselling services are insufficient at my institution	0.60	0.556	2.92 (1.17)
A2	My programme focuses on examinations rather than holistic professional development	0.65	0.611	2.84 (1.16)
A3	Role models and professional socialisation opportunities are lacking	0.66	0.626	2.81 (1.14)
	**Domain B: Career development constraints**			
B1	There is a gap between my training and the demands of clinical midwifery work	0.63	0.596	2.95 (1.16)
B2	I pay little attention to how employment trends will affect my career	0.54	0.499	2.55 (1.15)
B3	I worry that midwifery may not be essential to future society	0.69	0.618	3.45 (1.16)
B4	I am unfamiliar with the midwifery job market	0.68	0.632	2.89 (1.09)
B5	Insufficient academic qualifications may lead to unequal treatment in my career	0.69	0.657	3.44 (1.06)
B6	Poor academic performance may affect my future job prospects	0.50	0.527	3.63 (0.94)
B7	If employment conditions are difficult, I may lower my expectations	0.67	0.670	3.61 (1.00)
	**Domain C: Career exploration uncertainty**			
C1	Lack of experience may lead to unfair treatment at work	0.66	0.690	3.50 (0.97)
C2	I am unsure whether I am suited to midwifery work	0.75	0.702	3.28 (1.06)
C3	I am unsure how to access career-related information	0.79	0.742	3.16 (1.06)
C4	I am unclear about how to explore career development in midwifery	0.79	0.746	3.28 (1.05)
C5	I am unclear about the scope and requirements of midwifery practice	0.78	0.721	3.01 (1.07)
	**Domain D: Practical competence concerns**			
D1	I worry I lack the manual dexterity needed for midwifery	0.87	0.757	3.13 (1.10)
D2	I worry my midwifery knowledge base is insufficient	0.76	0.723	3.43 (1.00)
D3	I worry my clinical skills are not sufficiently practised	0.75	0.716	3.47 (0.99)
D4	I struggle to organise tasks efficiently, which may affect my practice	0.82	0.750	3.08 (1.04)
	**Domain E: Psychological resilience barriers**			
E1	Repeated setbacks in midwifery work might deplete my motivation	0.82	0.755	3.11 (1.04)
E2	I find it easy to give up when facing difficulties at work	0.75	0.738	2.78 (1.00)
E3	I do not know how to cope when experiencing difficulties or pressure at work	0.80	0.772	2.99 (1.00)
E4	I fear making mistakes, which may undermine my confidence	0.84	0.778	3.19 (1.03)
	**Domain F: Family and peer obstacles**			
F1	My family does not support my wish to work as a midwife	0.66	0.630	2.58 (1.06)
F2	My family hopes I will pursue a different career	0.59	0.597	2.60 (1.01)
F3	My family is overly involved in my career planning	0.54	0.541	2.52 (1.01)
F4	Negative feedback from peers about midwifery would weaken my commitment	0.77	0.746	3.05 (1.02)
F5	Peer choices of different career paths make me feel pressured	0.75	0.711	2.98 (1.04)
	**Domain G: Personal attribute concerns**			
G1	I worry I cannot communicate well with mothers and obstetricians	0.84	0.761	3.11 (1.05)
G2	I worry I cannot manage obstetric emergencies effectively	0.87	0.768	3.22 (1.06)
G3	I worry my physical and emotional stamina is insufficient for high-intensity work	0.85	0.744	3.13 (1.07)
G4	I worry I may miss abnormal signs in mothers, with serious consequences	0.82	0.728	3.23 (1.06)
G5	I worry poor emotional regulation may cause conflicts with patients or families	0.80	0.696	3.02 (1.08)
	**Overall scale: α = 0.968**,** ITC range = 0.499–0.778**			

Note. ITC = corrected item-total correlation. Factor loadings from maximum likelihood exploratory factor analysis, single-factor solution. All loadings ≥ 0.50

### Confirmatory factor analysis

Although EFA supported a unidimensional solution, a single-factor CFA model with items grouped within their seven theoretically derived content domains was specified to examine whether the hypothesised domain structure holds under confirmatory constraints. The initial model fit indices were (χ²(495) = 2341.25, χ²/df = 4.73, CFI = 0.824, TLI = 0.861, RMSEA = 0.087, SRMR = 0.082. Modification indices were examined, and six pairs of residual covariances were added sequentially. All six pairs involved items within the same content domain and were retained only where a substantive explanation could be provided: A1–A2 (both reflect perceived inadequacy of institutional career guidance), C3–C4 (both reflect uncertainty about accessing career-related information and pathways), D2–D3 (professional knowledge and clinical skill proficiency are conceptually proximate), E2–E3 (both reflect difficulty coping with setbacks and pressure), F4–F5 (both reflect peer influence on career choice), and G1–G2 (both reflect concerns about clinical performance under demanding conditions). The modified model demonstrated improved fit: χ²(489) = 2142.68, χ²/df = 4.38, CFI = 0.930, TLI = 0.917, RMSEA = 0.074, SRMR = 0.068. Standardized factor loadings ranged from 0.511 to 0.916, all exceeding the conventional threshold of 0.50. Internal consistency was excellent (Cronbach’s α = 0.968). CFA results are presented in Table 3.

**Table 3:** Standardized factor loadings from confirmatory factor analysis of the PCBS-MU

Domain	Item	Standardized loading (λ)
A. Institutional guidance deficit	A1	0.701
	A2	0.835
	A3	0.916
B. Career development constraints	B1	0.595
	B2	0.603
	B3	0.695
	B4	0.511
	B5	0.764
	B6	0.543
	B7	0.695
C. Career exploration uncertainty	C1	0.561
	C2	0.668
	C3	0.780
	C4	0.769
	C5	0.770
D. Practical competence concerns	D1	0.788
	D2	0.751
	D3	0.738
	D4	0.796
E. Psychological resilience barriers	E1	0.773
	E2	0.705
	E3	0.791
	E4	0.805
F. Family and peer obstacles	F1	0.825
	F2	0.817
	F3	0.654
	F4	0.593
	F5	0.611
G. Personal attribute concerns	G1	0.821
	G2	0.898
	G3	0.853
	G4	0.864
	G5	0.785

Note. A1∼A2, C3∼C4, D2∼D3, E2∼E3, F4∼F5, G1∼G2. All loadings are from the modified model

### Internal consistency and item quality

Cronbach’s α for the full 33-item scale was 0.968, indicating excellent internal consistency. Corrected item-total correlations ranged from 0.499 to 0.778 (mean = 0.682), all exceeding the recommended threshold of 0.30 [26]. No item, if deleted, would increase the overall α coefficient, confirming that all items contribute meaningfully to the scale. Item-level statistics are presented in Table 2.

### Group differences in PCBS-MU scores

Parenting style was the strongest demographic correlate of PCBS-MU scores (Kruskal-Wallis H(3) = 29.87, *p* < .001, η² = 0.035). Bonferroni-corrected pairwise comparisons (adjusted α = 0.0083) revealed that authoritative parenting was associated with significantly lower barrier scores than each of the other three styles: authoritarian (U = 10448, *p* < .001), permissive (U = 39861, *p* < .001), and uninvolved (U = 7120, *p* < .001). Median scores were lowest for authoritative parenting (Mdn = 99.0, IQR = [85.5, 112.0]) and highest for uninvolved parenting (Mdn = 110.0, IQR = [97.0, 123.0]). The three non-authoritative groups did not differ significantly from one another (all *p* > .20).

Rural students reported higher PCBS-MU scores than urban students (Mdn = 104.0 vs. 99.0, U = 62580, *p* = .002, *r* = .13). Sex, academic grade, clinical placement experience, only-child status, and student leadership role were not significantly associated with PCBS-MU scores (all *p* > .05). Group comparison results are summarised in Table 1.

### Regression analysis

Hierarchical linear regression with nine predictors (sex, grade, clinical experience, residence, only-child status, student leadership role, and three parenting style dummy variables) explained 4.3% of variance in PCBS-MU total scores (adjusted R² = 0.032, F(9, 757) = 3.78, *p* < .001). Parenting style was the only independent predictor reaching statistical significance. Compared with authoritative parenting, both authoritarian (B = 11.03, SE = 3.09, *p* < .001) and uninvolved parenting (B = 12.63, SE = 3.60, *p* < .001) were associated with substantially higher barrier scores; permissive parenting was also significant (B = 6.24, SE = 1.98, *p* = .002). Residence approached but did not reach significance (B = 3.21, SE = 1.92, *p* = .095). All VIF values were below 1.20, indicating no multicollinearity concerns. Regression results are presented in Table 4.

Spearman correlations, computed for descriptive purposes only, indicated that rural residence was positively associated with total PCBS-MU scores (rₛ = 0.111, *p* = .002). Grade, clinical experience, and sex showed no significant bivariate associations (all *p* > .15).

**Table 4:** Hierarchical linear regression: predictors of PCBS-MU total score (*N* = 767)

Predictor	B	SE	t	*p*	95% CI	VIF
Sex	0.91	4.40	0.21	0.836	[− 10.72, 11.54]	1.02
Grade	0.82	0.82	0.99	0.322	[− 0.80, 2.43]	1.08
Clinical exp	0.78	1.06	0.74	0.458	[− 1.29, 2.86]	1.08
Residence	3.21	1.92	1.67	0.095	[− 0.55, 6.97]	1.18
Only child	0.69	2.30	0.30	0.765	[− 3.82, 5.20]	1.16
Student leadership role	−0.10	1.88	−0.05	0.957	[− 3.80, 3.60]	1.02
Parenting: Authoritarian	11.03	3.09	3.57	< 0.001	[4.97, 17.09]	1.08
Parenting: Permissive	6.24	1.98	3.15	0.002	[2.35, 10.13]	1.14
Parenting: Uninvolved	12.63	3.60	3.51	< 0.001	[5.57, 19.69]	1.09

Model: R² = 0.043, Adjusted R² = 0.032, F(9, 757) = 3.78, *p* < .001

Note. Reference category for parenting style = authoritative. 95% CI = 95% confidence interval for B. VIF = variance inflation factor. All VIF < 1.20

## Discussion

This study developed and validated the PCBS-MU, to our knowledge, the first psychometrically evaluated measure of perceived career barriers for Chinese midwifery undergraduates. EFA supported a single-factor structure, and the scale demonstrated excellent internal consistency (α = 0.968) with strong item-level performance across all 33 items. The finding of a unidimensional structure, despite items spanning seven conceptually distinct content domains, is substantively informative: it suggests that midwifery students’ career barrier perceptions cohere around a single global appraisal of occupational difficulty, rather than representing separable, independent sources of concern. The high inter-domain correlations observed here (*r* = .42–0.79) are consistent with this interpretation. This coherence may also reflect the shared structural context that frames all career-related concerns for this population: a sequential, cross-qualification licensing pathway that positions competence demands, family pressure, and market uncertainty within a single occupational horizon — that of being a midwifery graduate without direct midwifery registration. This unidimensional structure was further supported by the CFA, in which the modified model demonstrated good fit (CFI = 0.930, RMSEA = 0.074), with all standardised factor loadings exceeding 0.50, providing confirmatory evidence for the scale’s structural validity.

As a prespecified exploratory secondary analysis, the finding that parenting style was the dominant predictor of barrier perceptions, both as the strongest group difference and the sole independent predictor in the regression model, is consistent with SCCT’s proposition that family socialisation environments shape career cognitions through their effects on internal psychological resources [18]. Students who reported authoritarian or uninvolved parenting backgrounds scored, on average, approximately 11–13 points higher on the PCBS-MU than those from authoritative backgrounds — a difference representing roughly 0.45–0.51 standard deviations. This effect size is educationally meaningful: students from less supportive parenting backgrounds perceive meaningfully elevated career barriers compared with their authoritative-parenting peers.

The overall regression model, however, explained only 4.3% of variance in barrier scores, suggesting that the demographic and family background variables examined here account for a modest proportion of individual differences in barrier perceptions. This points to the likely importance of psychological mediators — such as self-efficacy, career identity, and coping orientation — that were not assessed in the present study and warrant inclusion in future research. These findings align with a growing body of research linking parenting style to self-efficacy, resilience, and career-related coping in health profession students [15, 19]. Authoritarian and uninvolved parenting have consistently been associated with lower psychological capital and less adaptive responses to challenge in Chinese student populations [15]. From a practical standpoint, this pattern points to a potentially modifiable intervention target: psychological resources development programmes, particularly those delivered during the transition into clinical training, may be especially beneficial for students from less supportive family backgrounds. Structured mentoring, resilience-building curricula, and peer support networks represent evidence-aligned strategies for this purpose [27, 28].

Rural students reported higher barrier perceptions than urban students (rₛ = 0.111, *p* = .002), though this association did not survive adjustment in the multivariate model. This pattern is broadly consistent with international evidence that students from lower-resourced backgrounds and regions face structural disadvantages in accessing high-quality clinical experiences, career guidance, and professional networks [29]. The attenuation of the rural effect in the regression model — after accounting for parenting style — suggests that family socialisation is a stronger proximal predictor than geographic background. Nonetheless, regional inequities in training quality and career visibility likely remain an independent source of concern, and may warrant targeted policy attention in the form of cross-regional placement agreements and enhanced career counselling provision in institutions serving predominantly rural student populations [30, 31].

The absence of significant effects for academic grade, clinical placement experience, sex, only-child status, and student leadership role warrants comment. The null finding for grade is perhaps surprising, given theoretical expectations — grounded in professional identity development research [32, 33] — that barrier perceptions might peak during the clinical transition years before declining with accumulated competence. One explanation is that the cross-sectional design lacks the temporal resolution to detect such a trajectory; a longitudinal or experience-sampling design would be required to test this. The null finding for clinical placement experience may similarly reflect cohort effects, institutional variation in placement quality, or the insensitivity of a three-category variable to capture meaningful differences in students’ clinical exposure.

The PCBS-MU’s unidimensional structure offers practical advantages for educational and research use. A single total score is more straightforward to interpret than a multidimensional profile, and its excellent internal consistency means it can be used reliably in both research and routine monitoring contexts. The seven content domains, while not empirically distinct factors, nonetheless provide content-referenced guidance for curriculum design: institutions seeking to reduce specific facets of barrier perception — such as career exploration uncertainty or practical competence concerns — can use domain-level item subsets as process indicators to evaluate whether targeted interventions are achieving their intended effects.

### Limitations

Several limitations should be noted. First, the cross-sectional design precludes causal inference and cannot capture how barrier perceptions evolve as students progress through training; longitudinal designs would be better suited to test developmental hypotheses. Second, parenting style was assessed with a single item, which limits measurement precision; future studies should consider validated multi-item measures of parenting. Third, the sample was predominantly Han Chinese and, while spanning five geographic regions, was concentrated in East China (46% of participants), limiting generalisability to western, central, and other underrepresented regions, as well as to ethnic minority groups. Fourth, no convergent or discriminant validity evidence with external measures was collected; future validation studies should include established measures of self-efficacy, professional identity, and career commitment to strengthen the validity evidence base. Fifth, item generation relied on literature review and expert consultation alone; semi-structured interviews with midwifery undergraduates were not conducted, which may limit the ecological validity of the content domains. Sixth, content validity was assessed by a panel of six midwifery educators in a single round; future instrument refinement would benefit from a multidisciplinary panel including clinical midwives, career psychologists, and psychometricians, across multiple rounds of expert consultation. Finally, item wording assumed a Chinese educational context and labour market, which may limit the scale’s applicability in other cultural settings without further adaptation and validation.

### Implications for nursing education

The PCBS-MU provides midwifery educators with a brief, reliable, and contextually appropriate tool for monitoring career barrier perceptions among Chinese midwifery undergraduates. Its use in routine programme evaluation could help identify cohorts at elevated risk of attrition before career intentions crystallise. The strong association with parenting style points toward early screening and targeted support for students from less authoritative family backgrounds, particularly at the onset of clinical training. Institutional investment in psychological support services, structured mentoring, and career guidance is likely to yield the greatest benefit for this subgroup. Although the PCBS-MU was developed and validated in a Chinese context, the challenges it captures — including licensing pathway ambiguity, clinical competence anxiety, and family socialisation pressures — are not unique to China. The scale may serve as a template for adaptation in other settings where midwifery workforce retention is a concern, pending cross-cultural validation and measurement invariance testing.

## Conclusion

This study introduced and validated the PCBS-MU, a 33-item scale for measuring perceived career barriers in Chinese midwifery undergraduates, with EFA supporting a single dominant factor structure. The PCBS-MU demonstrated satisfactory initial psychometric properties, and an exploratory secondary analysis identified parenting style as the primary correlate of barrier perceptions, consistent with SCCT’s emphasis on family socialisation as a distal predictor of career cognitions. The PCBS-MU offers a practical and theoretically grounded instrument for researchers and educators seeking to monitor, understand, and address career barrier perceptions in this workforce-critical population.

## Data Availability

The datasets generated and/or analyzed during the current study are not publicly available due to participant confidentiality constraints but are available from the corresponding author on reasonable request.

## Abbreviations

CBI: Career Barriers Inventory
CVI: Content Validity Index
EFA: Exploratory Factor Analysis
I-CVI: Item-level Content Validity Index
IQR: Interquartile Range
KMO: Kaiser-Meyer-Olkin
PCBS-MU: Perceived Career Barrier Scale for Midwifery Undergraduates
SCCT: Social Cognitive Career Theory
SD: Standard Deviation
VIF: Variance Inflation Factor
WHO: World Health Organization
